# Pathophysiological mechanisms in severe preeclampsia: role of upregulated proteins in blood pressure, extracellular matrix and immunity

**DOI:** 10.61622/rbgo/2025rbgo54

**Published:** 2025-07-15

**Authors:** Caroline Cristina Pinto-Souza, Julyane Natsumi Saito Kaihara, Bruno César Rossini, Ricardo de Carvalho Cavalli, Lucilene Delazari dos Santos, Valéria Cristina Sandrim

**Affiliations:** 1 Universidade Estadual Paulista Instituto de Biociências de Botucatu Departamento de Biofísica e Farmacologia Botucatu SP Brasil Departamento de Biofísica e Farmacologia, Instituto de Biociências de Botucatu, Universidade Estadual Paulista, Botucatu, SP, Brasil.; 2 Universidade Estadual Paulista Faculdade de Ciências Agronômicas Departamento de Biotecnologia e Bioprocessos São Paulo Brasil Departamento de Biotecnologia e Bioprocessos, Faculdade de Ciências Agronômicas, Universidade Estadual Paulista, Botucatu, São Paulo, Brasil.; 3 Universidade de São Paulo Faculdade de Medicina de Ribeirão Preto Departamento de Ginecologia e Obstetrícia Ribeirão Preto SP Brasil Departamento de Ginecologia e Obstetrícia, Faculdade de Medicina de Ribeirão Preto, Universidade de São Paulo, Ribeirão Preto, SP, Brasil.; 4 Universidade Estadual Paulista Faculdade de Medicina de Botucatu Botucatu SP Brasil Instituto de Biotecnologia, Universidade Estadual Paulista, Botucatu, SP, Brasil; Programas de Pós-Graduação em Doenças Tropicais e em Pesquisa e Desenvolvimento (Biotecnologia Médica), Faculdade de Medicina de Botucatu (FMB), Universidade Estadual Paulista, Botucatu, SP, Brasil

**Keywords:** Pre-eclampsia, Severe features, Blood pressure, Plasma proteins, Hypertension, Extracellular matrix, Immunity

## Abstract

**Objective:**

This study aims to compare the plasma protein profiles between 7 preeclampsia patients with severe features (PE+) and 7 preeclampsia patients without severe features (PE-) and 10 healthy pregnancies (HP); identify differentially expressed proteins among these groups and explore the altered signaling pathways and their association with the severity of this cardiovascular condition.

**Methods:**

Plasma proteins were quantified using mass spectrometry, followed by comprehensive bioinformatics and statistical analyses. Protein identification and annotation were performed using UniProt and PatternLab for Proteomics. Multivariate statistical analyses, including PLS-DA and sPLS-DA, as well as VIP score evaluation and Volcano plot visualization, were conducted with MetaboAnalyst to assess group separation and identify key discriminative features. Functional enrichment and pathway analyses were carried out using Metascape.

**Results:**

Using a fold change and volcano plot validation of 1.2, comparisons between HP and PE+ revealed that proteins such as AMBP (inter-alpha trypsin inhibitor light chain), VTN (vitronectin), CLU (clusterin), F2 (prothrombin), and PZP (pregnancy zone protein) were upregulated in PE+. Conversely, ITIH4 (inter-alpha trypsin inhibitor heavy chain H4), APOL1 (apolipoprotein 1) and SERPIND1 (heparin cofactor II) were downregulated in PE+ relative to HP. When comparing HP with PE-, SERPINA3 (alpha-1-antichymotrypsin) and HBB (hemoglobin subunit beta) were downregulated in PE-. Between PE- and PE+, APCS (serum amyloid P component) and HBB were upregulated in PE+; whereas SERPINC1 (antithrombin), PSG1 (pregnancy-specific beta-1-glycoprotein 1), ITIH4, and C5 (complement C5) were downregulated in PE+ compared to PE-.

**Conclusion:**

These findings offer valuable insights into the different pathophysiological mechanisms underlying the two subgroups of PE. The upregulated proteins in PE+ (AMBP, VTN, CLU, F2, PZP, APCS, and HBB) play key roles in regulating blood pressure, modulating the extracellular matrix and influencing immune responses. Overall, this research deepens our understanding of the complexity and clinical significance of PE.

## Introduction

Preeclampsia (PE) is a hypertensive disorder diagnosed after the 20th week of pregnancy, defined by blood pressure readings exceeding 140/90 mm Hg and characterized by target organ damage (kidney injury, liver dysfunction and lung complications).^(1)^

This disease is related to several maternal (cerebral or visual disturbances) and perinatal (neurological problems, premature birth) implications, in addition to presenting increased cardiovascular risk and requiring high costs of obstetric and hospital medical care.^(1)^

This cardiovascular damage also comes with endothelial dysfunction; oxidative stress impairment and hyperlipidemia.^(2,3)^ Although the available literature describes that delivery is the main resolution, antihypertensive agents should be administered to improve clinical outcomes. Similar pathophysiological characteristics were joined into certain phenotypes, to support the appropriate management. Also, there is more than one factor that contributes to its development (multifetal gestation, renal and autoimmune diseases, type 1 or type 2 diabetes mellitus and chronic hypertension).^(1)^

According to the presence and intensity of signal and symptoms, PE patients are classified into non-severe (PE-) and severe cases (PE+),^(1)^ the latter being developed for more than 50% of the patients.^(4)^ Features differentiating these subgroups are blood pressure of or higher than 160/110 mmHg and organ failure. This includes increase in liver enzymes twice as high as normal; signs of central nervous system dysfunction (altered vision and mental status, headache, cerebrovascular accident); thrombocytopenia (< 100 000/mm^3^) and intrauterine growth restriction (IGR), which happens when a fetus is unable to achieve its natural growth potential at any stage of gestation due to compromised placental function.^(4,5)^ Fetuses with IGR are at higher risk for perinatal complications, including morbidity and mortality, as well as long-term health issues, such as developmental delays in neurological and cognitive abilities.^(6)^

The verification of IGR is achieved through the Delphi method, an iterative technique in which experts score a series of standardized questions on the subject. These responses are then reviewed, sent back to the participants, and the process is repeated in multiple rounds with increasing detail until consensus is reached. ^(5,7)^ As a result, it helps better identify fetuses at risk and reduces the over-diagnosis of physiological smallness.^(5,7)^ Additionally, a study reached a consensus on a definition of IGR, based on five key points.^(6)^ First, a distinction was made between early and late IGR, with 32 weeks of gestation serving as the cutoff. Second, it was agreed that the presence of congenital anomalies should be excluded. Third, absolute size measurements were set at lower thresholds (3rd percentile) compared to the more commonly used 10th percentile. Fourth, functional parameters were incorporated into the definition, either as standalone factors (such as absent end-diastolic flow in the umbilical artery) or contributory indicators (including umbilical artery-pulsatility index or uterine artery-pulsatility index > 95th percentile, and cerebroplacental ratio < 5th percentile).^(6)^

Based on recent guidelines, proteinuria is unessential for the diagnosis and severity evaluation of the disease.^(8)^ Higher protein excretion does not appear to be related to poor maternal or neonatal outcomes, and monitoring proteinuria may result in unnecessary preterm deliveries and associated neonatal complications.^(8)^ Suspecting that a woman developed the PE+, her hospitalization is required to monitor progression of the disease as well as to promote quick intervention.^(4)^ Our research group has previously investigated the severity subgroups^(9–11)^ in the matter of circulating arginase 2, plasma endothelial nitric oxide synthase and myeloperoxidase activity and concentrations, respectively.

Furthermore, although the experimental evidence of proteomics in the field of PE is growing, most of the published articles analyses the onset^(12–14)^ and prediction of the disease.^(15,16)^

Also, we have recently found proteins that are differentially expressed between responsive and non-responsive PE patients,^(17)^ proposing that they demonstrate a complex interaction between inflammatory, immune, and metabolic processes, as well as their relationship with the responsiveness to antihypertensive therapy and the disease's pathophysiology.

Moreover, this work explores PE+ and PE- with a proteomics view, as this methodology promotes a wide and objective plasma protein analysis, acting as an advantageous tool to search potential biomarkers. Therefore, the differentially expressed molecules could promote early diagnosis with clinical validity. In view of the lack of studies regarding severity subgroups and untargeted proteomics, an almost complete proteomic coverage, considered a reliable technology for biomarker discovery by the scientific community,^(18–22)^ we intended to verify not only the profile database, but also, to search for any correlation between them with biological processes and pathways involved with PE.

Thereby, we intended 1) to compare the proteins profile in PE versus HP; 2) to investigate those that are differently expressed in the PE+ and PE- to the HP controls, in view of this, 3) to identify the signaling pathways altered between PE subgroups and determine their association with the pathophysiological aspects of this hypertensive disorder. Thus, to better understand the severity subgroups and search potential biomarkers for the prevention and the treatment of the disease.

## Methods

We selected a total of 14 patients with PE, including 7 with severe features (PE+) and 7 without severe features (PE-), along with 10 healthy controls (HP). All participants were enrolled consecutively at the Department of Gynecology and Obstetrics, University Hospital of FMRP-USP. Informed consent was obtained from all participants. The diagnosis of PE was made according to the ACOG Practice Bulletin.^(1)^

Patients were classified as having PE if they exhibited a systolic blood pressure greater than 140 mmHg and diastolic pressure greater than 90 mmHg on two separate measurements taken 4 hours apart while at rest, along with proteinuria greater than 0.3 g/L in a 24-hour urine sample. In the absence of proteinuria, PE was diagnosed in the presence of hypertension together with one or more of the following conditions: thrombocytopenia (platelet count < 100,000 × 10^9^/L), elevated liver enzyme levels (twice the normal concentration of liver transaminases), renal impairment, pulmonary edema, and cerebral or visual disturbances.^(1)^ Women with preexisting hypertension, whether complicated by superimposed PE, were excluded from the study. Among the volunteers who consented to participate, 15 mL of venous blood was collected using standard procedures.

The samples were collected in Vacutainer tubes (Becton-Dickinson) containing EDTA, then centrifuged at room temperature for 10 minutes at 3200×g. Aliquots of 250 μL were separated and stored with whole blood at −80°C until analysis. Plasma samples were subsequently used for biochemical and proteomic analysis.

According to the guidelines outlined in the ACOG Practice Bulletin^(1)^ the following criteria were used to classify PE with severe features:

- Systolic blood pressure of 160 mm Hg or higher, or diastolic blood pressure of 110 mm Hg or higher, on two separate occasions at least 4 hours apart (unless antihypertensive treatment is initiated prior to this time);
- Thrombocytopenia (platelet count below 100,000 x 10^9^/L);
- Impaired liver function, as evidenced by elevated blood liver enzyme levels (twice the normal upper limit), along with severe, persistent right upper quadrant or epigastric pain that does not respond to medication and cannot be explained by other diagnoses;
- Renal insufficiency, indicated by a serum creatinine concentration greater than 1.1 mg/dL, or a doubling of serum creatinine in the absence of other renal diseases.

Briefly, the samples were prepared by combining 2 μL of each crude sample with 98 μL of ammonium bicarbonate (Ambic, Sigma-Aldrich, San Luis, CA, USA). This ratio was scaled up by a factor of 7.5, resulting in a mixture of 15 μL of each sample with 735 μL of Ambic, reaching a final volume of 750 μL (1:50). This dilution was optimized for protein quantification, electrophoresis, and digestion. To concentrate the plasma blood, a sample homogenizer and centrifugation were used. Once prepared, the samples were stored in a freezer until further use.^(17)^

Additional information about protein quantity and quality; enzymatic digestion; peptide sequencing by mass spectrometry, and proteomic data analysis, focusing on univariate and chemometric techniques using MetaboAnalyst software, can be read in our previous paper.^(17)^ Furthermore, the original mass spectrometry data presented in the study are openly available in the MassIVE Repository from Computer Science and Engineering University of California, San Diego (https://massive.ucsd.edu/, accessed on 22 July 2024) with the dataset identifier: MSV000095404, and doi: 10.25345/C50V89V22).^(17)^

Pathway and Process Enrichment Analysis were explored through the MetaScape tool^(23)^ and various ontology sources, including KEGG Pathway, GO Biological Processes, Reactome Gene Sets, Canonical Pathways, CORUM, WikiPathways and PANTHER Pathway were applied. The entire genome was used as the background for enrichment analysis.^(23)^

Terms with a p-value < 0.01, a minimum count of 3, and an enrichment factor > 1.5 (where the enrichment factor represents the ratio of observed counts to those expected by chance) were collected. These terms were then grouped into clusters based on their similarities.^(23)^ P-values were calculated using the cumulative hypergeometric distribution,^(24)^ while q-values were computed using the Benjamini-Hochberg procedure to adjust for multiple testing.^(25)^ Kappa scores were used as a similar metric for hierarchical clustering of enriched terms.^(26)^ Clusters with a similarity greater than 0.3 were considered significant, with the most statistically significant term in each cluster chosen to represent it.

Several databases such as STRING,^(27)^ BioGrid,^(28)^ OmniPath, and InWeb_IM^(29)^ were applied to verify protein-protein interactions and only the physical associations were considered in STRING (with a physical score > 0.132) and BioGrid.

Therefore, the resulting network contains proteins that exhibit physical interactions with at least one other protein in the gene list. If the network contained between 3 and 500 proteins, the Molecular Complex Detection (MCODE) algorithm^(30)^ was applied to identify densely connected components. Pathway and process enrichment analysis was conducted on each MCODE element independently. The three most statistically significant terms, based on p-value, were retained as the functional description for each component and are presented in tables below the corresponding network plots.^(23,30)^

Clinical characteristics of the HP group and women with PE+ or PE- were compared using an unpaired t-test or One-Way ANOVA, followed by Tukey's multiple comparison tests for parametric data. For nonparametric, the Kruskal-Walli's test was used, followed by Dunn's multiple comparison tests. Categorical variables were compared using χ^2^ tests. Statistical analyses were conducted using GraphPad Prism 8.0 (San Diego, CA, USA). A p-value < 0.05 was considered statistically significant.

The study was conducted in compliance with the Declaration of Helsinki and received approval from the Research Ethics Committee at Ribeirao Preto Medical School, University of São Paulo 5147382 (FMRP-USP, *Certificado de Apresentação de Apreciação Ética:* 37738620.0.0000.5440).

## Results

Table 1 presents a summary of the characteristics of HP women and PE patients, categorized into those with severe features (PE+) and those without severe features (PE-). Compared to the HP group, both PE subgroups had higher body mass index (BMI), as well as elevated diastolic and systolic blood pressures. The PE+ subgroup had lower gestational age at delivery and newborn weight in relation to the control group. When comparing the two disease subgroups, PE+ showed a lower BMI, gestational age at delivery and newborn weight, whereas exhibiting higher urea concentrations than PE- (all p < 0.05).

**Table 1:** Clinical characteristics of the participants included in this study

Parameters	HP	PE-	PE+	p-value
Age (years)	25.1 ± 5.1	26.9 ± 5.6	32.1 ± 6.9	0.0738
BMI (kg/m²)	26.0 (23.2 - 28.0)	36.5 (35.0 - 37.6)*#	31.2 (28.2 - 32.6)*	< 0.0001
SBP (mmHg)	101.5 (92.5 - 110.0)	140.0 (130.0 - 160.0)*	143.0 (127.5 - 161.8)*	0.0001
DBP (mmHg)	69.5 (60.0 - 77.5)	80.0 (70.0 - 100.0)*	90.0 (87.5 - 94.0)*	0.0070
HR (beats per min)	80.0 (80.0 - 86.3)	80.0 (80.0 - 92.0)	81.0 (80.0 - 85.0)	0.8966
Hemoglobin (g/dL)	11.6 (10.9 - 14.1)	10.8 (10.1 - 11.4)	11.9 (11.4 - 13.3)	0.1097
Hematocrit (%)	34.6 (33.0 - 43.1)	32.0 (30.0 - 34.0)	36.0 (34.5 - 39.0)	0.0971
Creatinine (μmol/L)	NA	0.7 (0.6 - 0.9)	0.9 (0.7 - 1.0)	0.2150
Urea (mg/dL)	NA	17.0 (12.0 - 21.0)#	24.0 (20.0 - 33.0)	0.0441
Proteinuria (mg/24h)	NA	374.0 (362.0 - 865.0)	2155.0 (630.4 - 5852.0)	0.113
GA at sampling (weeks)	38.0 (33.0 - 39.5)	37.5 (28.0 - 38.5)	29.5 (27.8 - 33.3)	0.0649
GA at delivery (weeks)	39.0 (39.0 - 40.0)	38.0 (37.0 - 39.0)#	32.0 (27.8 - 34.8)*	< 0.0001
Newborn weight (g)	2790.0 (2720.0 - 3580.0)	3260.0 (2920.0 - 3900.0)#	1215.0 (905.0-2040.0)*	< 0.0001

* p < 0.05 vs HP;

# p < 0.05 vs PE+. Data are expressed as mean ± standard error of the mean, median (25th–75th percentile) or percentage. DBP: Diastolic blood pressure; GA: Gestational age; HR: Heart rate; NA: not available; SBP: Systolic blood pressure. HP, healthy pregnancy; PE-, preeclampsia without severe features; PE+, preeclampsia with severe features

**Figure 1:**
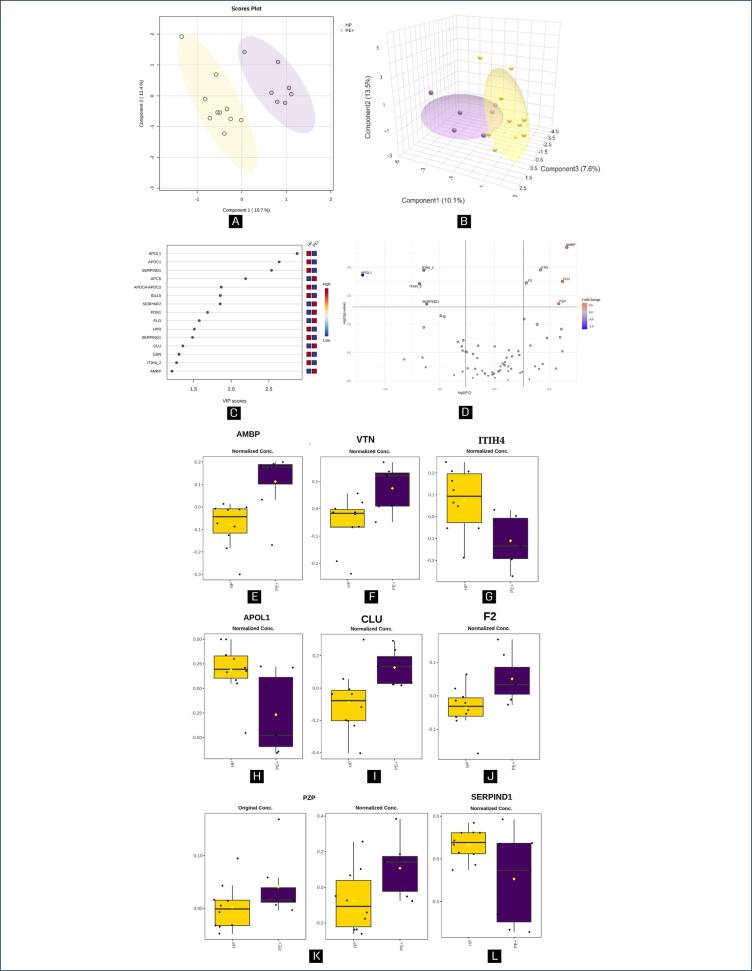
Chemometric and univariate statistical analyses of the eight proteins identified in the plasma of healthy pregnancy (HP; in yellow) and PE patients with severe features (PE+; in purple) using MetaboAnalyst

**Figure 2:**
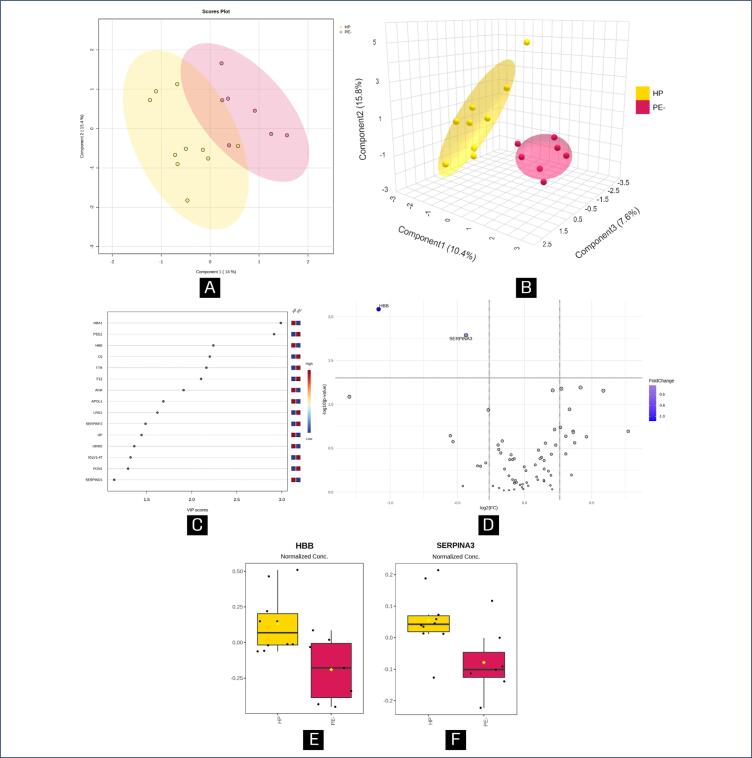
Chemometric and univariate statistical analyses of plasma proteins from healthy pregnancy (HP; yellow) and PE patients without severe features (PE-; pink) using MetaboAnalyst

**Figure 3:**
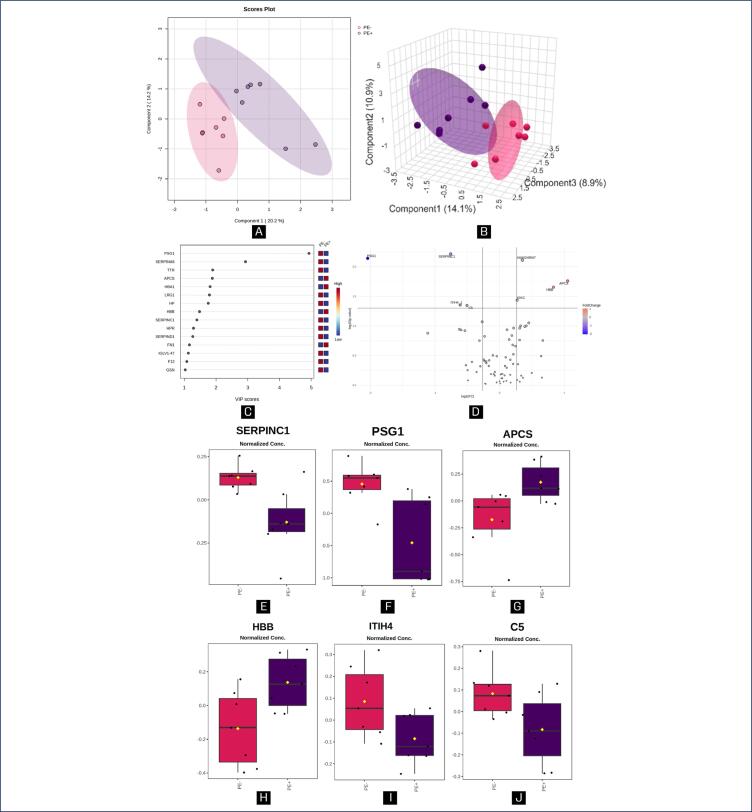
Chemometric and univariate statistical analyses of plasma proteins from PE patients with non-severe features (PE-; pink) and severe features (PE+; purple) using MetaboAnalyst

Figures 1–3 illustrate the statistical methods applied to analyze the data related to the proteins differentially expressed in the following group comparisons using MetaboAnalyst software: HP versus PE+ (Figure 1), HP versus PE- (Figure 2), and PE- versus PE+ (Figure 3). Chemometrics analysis using Partial Least Squares Discriminant Analysis (PLS-DA) was employed to extract meaningful patterns. The 2-dimensional PLS-DA differentiated the groups based on the protein dataset using scores from the first two components: (10.7%; 13.4%) for Figure 1a, (14.0%; 15.4%) for Figure 2a, and (20.2%; 14.2%) for figure 3a. A 3-dimensional sparse PLS-DA (sPLS-DA) further showed discrimination between the groups using scores from three components: (10.1%; 13.5%; 7.6%) for figure 1b, (10.4%; 15.8%; 7.6%) for Figure 2b and (14.1%; 10.9%; 8.9%) for Figure 3b. Univariate Statistical Analysis was conducted to examine the relationship between protein-related data (intensity peaks) and the plasma samples through t-tests, aiding in the understanding of protein distribution, variability and significance.

(A) 2-dimensional partial least squares discriminant analysis (PLS-DA) score plot. (B) 3-dimensional sparse partial least squares discriminant analysis (sPLS-DA) score plot. (C) Variable importance in projection (VIP) plot showing the proteins that most significantly contribute to the differentiation between HP and PE+, as determined by PLS-DA. (D) Volcano plot highlighting eight differentially expressed proteins between HP and PE+: five upregulated (AMBP, VTN, CLU, F2 and PZP; all > in PE+) and three downregulated (ITIH4, APOL1, and SERPIND1; all < in PE+). The red and blue boxes on the right side indicate the relative abundance of each protein in the respective groups. Boxplots of the eight differentially expressed proteins in the plasma of HP women and PE+ patients: (E) AMBP (inter alpha trypsin inhibitor light chain); (F) VTN (vitronectin); (G) ITIH4 (inter alpha trypsin inhibitor heavy chain H4); (H) APOL1 (apolipoprotein L1); (I) CLU (clusterin); (J) F2 (prothrombin); (K) PZP (pregnancy zone protein), and (L) SERPIND1 (heparin cofactor II). HP, healthy pregnancy; PE+, preeclampsia with severe features

(A) 2-dimensional partial least squares discriminant analysis (PLS-DA) score plot. (B) 3-dimensional sparse partial least squares discriminant analysis (sPLS-DA) score plot. (C) Variable importance in projection (VIP) scores, showing the proteins that contribute most to the differentiation between HP and PE-, as identified through PLS-DA. (D) Volcano plot illustrating two downregulated proteins in PE- compared to HP: HBB (hemoglobin subunit beta) and SERPINA3 (both < in PE-). Red boxes on the right indicate the relative amounts of each protein in the respective groups. Boxplots of the two differentially expressed proteins in the plasma of HP women and PE- patients: (E) HBB and (F) SERPINA3. HP, healthy pregnancy; PE-, preeclampsia without severe features

(A) 2-dimensional partial least squares discriminant analysis (PLS-DA) score plot. (B) 3-dimensional sparse partial least squares discriminant analysis (sPLS-DA) score plot. (C) Variable importance in projection (VIP) scores showing the proteins that contribute most to the differentiation between PE+ and PE-, as identified through PLS-DA. (D) Volcano plot illustrating six differentially expressed proteins when comparing PE+ with PE-. Two proteins were upregulated (APCS and HBB all > in PE+) and four proteins were downregulated (SERPINC1, PSG1, ITIH4, and C5; all < in PE+). Red and blue boxes on the right indicate the relative amounts of each protein in the respective groups. Boxplots of the differentially expressed proteins in the plasma of PE- and PE+ patients: (E) SERPINC1 (antithrombin III); (F) PSG1 (pregnancy-specific beta-1-glycoprotein 1); (G) APCS (serum amyloid P component); (H) HBB (hemoglobin subunit beta); (I) ITIH4 (inter-alpha trypsin inhibitor heavy chain H4), and (J) C5 (complement component 5). PE-, preeclampsia without severe features; PE+, preeclampsia with severe features

The Variable Importance in Projection (VIP) score identifies the most influential proteins contributing to class separation, ranked by their ability to distinguish HP from PE+ (Figure 1c), HP from PE- (Figure 2c) and PE- from PE+ (Figure 3c). Proteins further from the Y-axis have made a greater contribution to the classification. Volcano plots (Figure 1d, 2d, and 3d) highlight biologically and statistically significant features, based on fold change (x-axis) and test statistics (y-axis). Figure 1a shows the PLS-DA analysis, indicating that 10.7% of the total variation between the HP and PE+ groups were explained by the first component, while 13.4% of the within-group variation were attributed to the second component, demonstrating that there was a full separation between the groups.

The sPLS-DA analysis (Figure 1b) demonstrated that component 1 accounted for 10.1% of the total between-group variation, component 2 explained 13.5%, and component 3 contributed 7.6%, suggesting a slight overlap between the groups. In figure 1c, the top four proteins contributing most to the differentiation between the HP and PE+ identified through PLS-DA were APCS (serum amyloid P component), SERPIND1 (heparin cofactor II), APOC1 (apolipoprotein C1), and APOL1 (apolipoprotein L1), all with VIP scores greater than 2.0.

The volcano plot in figure 1d revealed eight differentially expressed proteins between HP and PE+. Five proteins were upregulated in PE+: AMBP (inter alpha trypsin light chain), VTN (vitronectin), CLU (clusterin), F2 (prothrombin), and PZP (pregnancy zone protein). While three were downregulated, ITIH4 (inter alpha trypsin inhibitor heavy chain H4), APOL1, and SERPIND1). Figures 1e–l show the normalized peak intensities for the following proteins: AMBP, VTN, ITIH4, APOL1, CLU, F2, PZP, and SERPIND1, all of them exhibited a statistically significant difference (p < 0.05).

The PLS-DA analysis comparing HP and PE- showed that the first component accounted for 14.0% of the total between-group variation, while the second component explained 15.4% of the within-group variation (Figure 2a) and demonstrated a minimal overlap between them. Nevertheless, in figure 2b, the sPLS-DA analysis revealed that component 1 explained 10.4% of the total variation between the groups, component 2 accounted for 15.8%, and component 3 contributed 7.6%, indicating a complete separation of the groups. Figure 2c highlights the top six proteins that contributed most to the differentiation between HP and PE-, as identified through PLS-DA with VIP scores greater than 2.0: F12 (coagulation factor XII), TTR (transthyretin), C6 (complement component 6), HBB (hemoglobin subunit beta), PSG1 (pregnancy-specific beta-1-glycoprotein 1), and HBA (hemoglobin subunit alpha). The volcano plot in figure 2d identified two differentially expressed proteins between HP and PE-, both downregulated (HBB and SERPINA3 all < PE-). Figures 2e–f show the normalized peak intensity data for HBB and SERPINA3, respectively, both showed a statistically significant difference (p < 0.05).

Chemometric statistical analyses of the proteomics data using 2-dimensional PLS-DA revealed that the first component accounted for 20.2% of the total variation between PE- and PE+, while the second component explained 14.2% of the within-group variation (Figure 3a), with a slight intersection. Furthermore, 3-dimensional sPLS-DA indicated that component 1 accounted for 14.1% of the total between-group variation, component 2 contributed 10.9%, and component 3 accounted for 8.9% (Figure 3b), showing clear separation between the groups. Figure 3c highlights the top two proteins—SERPINA6 (corticosteroid-binding globulin) and PSG1 (pregnancy-specific beta-1-glycoprotein 1)—that contributed most to the differentiation between PE- and PE+ as identified through PLS-DA, with VIP scores greater than 2.0.

The volcano plot in figure 3d identifies six differentially expressed proteins between PE- and PE+, with two upregulated (APCS and HBB; all > in PE+) and four downregulated (SERPINC1, PSG1, ITIH4, and C5; all < in PE+). Figures 3e–j display the normalized peak intensity data for SERPINC1, PSG1, APCS, HBB, ITIH4, and C5, respectively, all of them demonstrated a statistically significant difference (p < 0.05).

**Figure 4:**
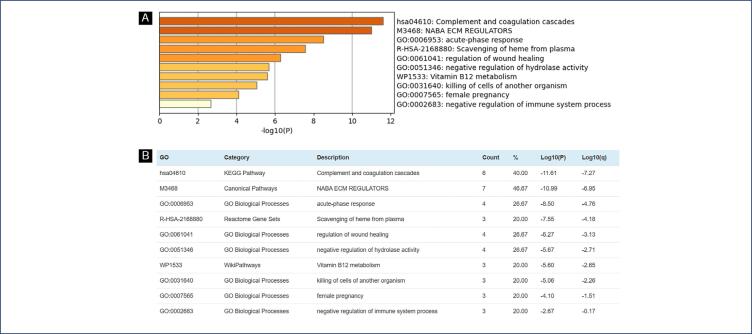
Bar Chart Showing Statistically Enriched Terms in Homo sapiens Preeclampsia Gene Sets, with p-Value Gradients (Metascape)

To investigate the key biological processes associated with the differentially expressed proteins between the groups, we used MetaScape^(23)^ for functional analysis (Figure 4a). First, we identified all statistically enriched terms, including Gene Ontology (GO) and Kyoto Encyclopedia of Genes and Genomes (KEGG) terms, based on the default settings in Express Analysis.^(23)^ We also calculated accumulative hypergeometric p-values and enrichment factors for filtering purposes.^(24,26)^ The remaining significant terms were hierarchically clustered into a tree using Kappa-statistical similarities based on their gene memberships. A Kappa score threshold of 0.3 was applied to aggregate the terms into clusters. Next, for each gene list, pathway and process enrichment analyses were performed using the following ontology sources: KEGG Pathway, GO Biological Processes, Reactome Gene Sets, Canonical Pathways, CORUM, WikiPathways, and PANTHER Pathway (Figure 4b).^(23)^ All genes in the genome were used as the background for enrichment. Terms with a p-value < 0.01, a minimum count of 3, and an enrichment factor > 1.5 were selected and grouped into clusters based on gene membership similarities. The most statistically significant term within each cluster was chosen to represent the cluster.^(23)^

(A) Ten representative clusters are shown, each labeled with a key enriched term that characterizes the cluster. (B) "Count" indicates how many genes from the input list are associated with the specific ontology term. "%" represents the proportion of input genes (considering only those annotated with at least one ontology term). "Log10(P)" corresponds to the base-10 logarithm of the enrichment p-value, and "Log10(q)" refers to the base-10 logarithm of the p-value adjusted for multiple testing. The term "NABA ECM" is associated with genes encoding enzymes and regulatory proteins involved in remodeling the extracellular matrix.

Working towards investigating all protein-protein interactions (PPI), the input genes were extracted from the PPI data source and used to construct a network. GO enrichment analysis was then applied to the network to identify the biological meanings.^(23,27–29)^ Subsequently, the Molecular Complex Detection (MCODE) algorithm was employed to detect densely connected neighborhoods of proteins within the network.^(30)^

## Discussion

This work explored the profile of proteins found in plasma samples in patients with PE, a global health problem, considering the severity diagnosis, in addition to identifying which of them would be differentially expressed between the subtypes of the disease and associated with its pathophysiology. Moreover, we enriched the analysis by using MetaScape to verify if the proteins could act in any pathway related to endothelial dysfunction. Therefore, we aimed to search novels biomarkers that propose alternatives for the treatment and prevention of PE.

Considering a fold change and volcano plot validation of 1.2, which helps to identify features that are both biologically and statistically significant, when comparing HP and PE+, there were eight proteins expressed differently; AMBP, VTN, CLU, F2, and PZP were all upregulated (> PE+); while SERPIND1, ITIH4, and APOL1 were downregulated (< PE+). In relation to HP versus PE-, there were two proteins differentially expressed: the SERPINA3 and HBB, both downregulated (< PE-). In PE- versus PE+, APCS and HBB were upregulated (all > PE+). On the other hand, SERPINC1, PSG1, ITIH4 and C5 were downregulated (all < PE+).

Most of the proteins differentially expressed between groups integrate coagulation and coagulate cascades, remodeling of the extracellular matrix, and inflammatory processes as seen in figure 4, suggesting that these signaling pathways could be modulated to prevent or treat the clinical outcomes of the hypertension syndrome. PE has already been explored by proteomics methods, mainly in the following subjects: prediction;^(15,16)^ differentiation between early and late onset^(12–14)^ and previous reviews.^(31,32)^ Nevertheless, few works had already explored the plasma protein profile in PE considering the disease severity classification. Thus, this study brings highlights about potential candidates for screening the signs and symptoms of the disease.

Features of PE+ include hypertension, central nervous system dysfunction, hepatocellular and pulmonary injuries, besides IGR.^(1,4)^ Therefore, this delicate condition necessitates an interdisciplinary health services team to provide appropriate prenatal care and ensure safe delivery.^(4)^

The upregulation observed in AMBP (> PE+ versus HP) (Figure 1e), an antioxidant and tissue repair protein with reductase, heme-binding and radical-scavenging activities^(33)^ (Figure 4), diverges from what has already been reported in the literature, as AMPB counteracts oxidative damage at blood-placenta interface, preventing leakage of free fetal hemoglobin into the maternal circulation.^(34)^ At the same time, other study described elevated levels of AMBP in cerebrospinal fluid of women with PE compared to normotensive pregnant.^(35)^ Thus, the higher plasma concentration of this protein in our data may be a compensatory mechanism related to the pathophysiology of the disease and more research regarding blood samples are needed to explore AMPB's biological processes implicated in hypertensive disorders.

**Figure 5:**
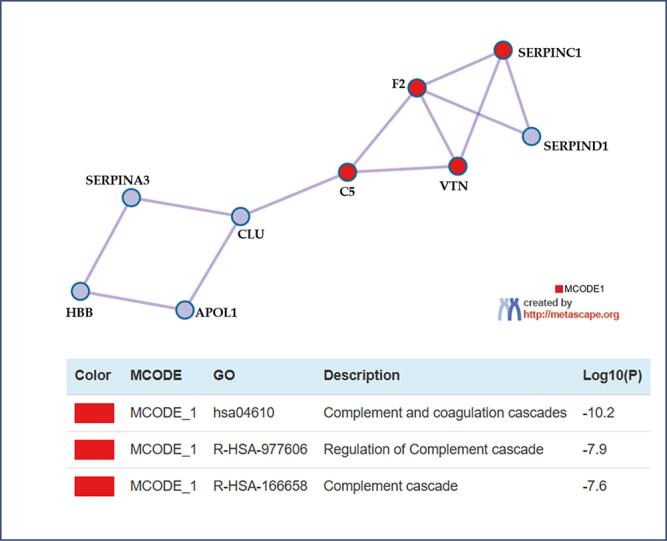
Network Visualization of Interacting Proteins and MCODE Components from Gene Lists in Preeclampsia (*Homo sapiens*, Metascape)

APOL1, apolipoprotein 1; C5, complement component C5; CLU, clusterin; F2, prothrombin; HBB, hemoglobin subunit beta; MCODE, Molecular Complex Detection; SERPINA3, alpha-1-antichymotrypsin; SERPINC1, antithrombin III; SERPIND1, heparin cofactor II; VTN, vitronectin

Interestingly, together with SERPINC1, a plasma protease inhibitor, F2, CLU and VTN showed significant interactions in complement and coagulation cascades (Figure 5), implying that they might modulate this pathway and contribute to the severity features of the disease. It has already been reported that augmented levels of F2 are associated with thrombophilia – a risk factor for pregnancy loss.^(36)^ Furthermore, a meta-analysis described that the F2 G20210A single-nucleotide polymorphism was associated with an increased predisposition for developing PE.^(37)^ Conversely, a group of authors showed that different phenotypes of the hypertensive disorder (early onset, severe features and low placental weight) were associated with thrombophilia factors such as anti-phospholipid antibodies, protein S deficiency, and hyperhomocysteineamia, in this order.^(38)^ Both works agree with the upregulation observed in the PE+.

In addition, several studies have already pointed out the upregulation of CLU blood concentrations in PE compared with controls.^(31,39,40)^ CLU is recognized as a multifunctional protein with an increased expression seen in renal, neurodegenerative, and cardiovascular diseases and cancers.^(39,41)^ One possible explanation for its elevated levels in PE+ could be endothelial cell dysfunction, potentially resulting from oxidative stress likely triggered by heat shock proteins—since CLU may function similarly—along with renal dysfunction, associated with glomerular endotheliosis and proteinuria, both of which are characteristic of hypertensive syndrome.^(39)^

Regarding VTN, it is a glycoprotein found in plasma, platelets, and extracellular matrix, which modulates cell adhesion and complement regulation, besides playing a key role in blood coagulation and fibrinolytic systems.^(42)^ To our knowledge, in scientific literature, there are few data exploring plasma VTN in the field of PE.^(43–45)^ Disagreeing with our results, plasma VTN levels were decreased in PE.^(43,44)^ On the contrary, a group of researchers indicated that 75-kDa single-chain VNT molecule increases 1.6 to 1.9-fold in plasma of patients with PE, therefore, indicating that this protein profile may be useful as an early marker of the disease.^(45)^

Furthermore, PZP is an immunomodulatory glycoprotein secreted by immune cells and has also been described in the placenta^(46,47)^ and it has already been reported that its blood levels are markedly enhanced in pregnancy, which suggests its fundamental roles during human gestation.^(48)^ Opposite to our data, one study showed that the PZP presents lower serum levels in PE patients in relation to healthy samples.^(21)^ However, its upregulation in a wide range of other inflammatory states is consistent with PZP having generalized functions as immunomodulators or stress responders.^(48)^

Moreover, along with other proteins, PZP protein was identified as pivotal to causal roles in the development of PE, guiding for the exploration of targeted therapies for the disease.^(49)^

SERPIND1, a thrombin inhibitor, on the other side, was downregulated in PE+ compared to the HP group (Figure 1l). This result aligns with previous studies,^(50)^ suggesting that SERPIND1 may play a role in inhibiting fibrin generation in the human placenta. Its reduced expression in PE could contribute to abnormal coagulation and placental dysfunction.^(50)^

In relation to HP versus PE-, there were two proteins differentially expressed: the SERPINA3 and HBB, both downregulated (< PE-) – therefore they may present protective effects to the endothelium. A group of authors suggested that SERPINA3 is involved in placental diseases, through its regulation by epigenetic, genetic and transcription factors-mediated actions.^(51)^ This protein integrates a superfamily which has function in maintaining body homeostasis as most of them inhibit the proteases activity. SERPINA3 is a typical acute-phase protein secreted into circulation during acute and chronic inflammation.^(51)^ Its major target is probably the neutrophil cathepsin G, a pro-inflammatory enzyme that plays roles in wound repair, platelet aggregation, extracellular matrix remodeling and apoptosis.^(51)^

Plasma levels of SERPINA3 are increased in women with PE and/or IGR,^(52,53)^ however in the context of acute myocardial infarction, it has been demonstrated that increased circulating levels of this protein is significantly associated with the risk of major adverse cardiovascular events.^(54)^

Blood hemoglobin (HB) is crucial for providing iron during pregnancy, a key nutrient for both maternal and fetal development. Still, studies have shown that HB levels exceeding 120 g/dL at the end of the second trimester are linked to a risk of PE and IGR that is less than three times greater.^(55,56)^ As well, since HBB plays a more critical role in HB's oxygen-carrying function compared to the alpha subunit,^(57)^ this may account for the upregulation observed in HP. The sample size in this study was relatively small, and further validation with a larger-scale study is recommended.

Moreover, depending on the gestational age at the time of sampling and the tissue studied, different results may emerge. Therefore, we encourage more proteomic research in other biological matrices to identify biomarkers that are most predictive of progression to severe forms of PE. It is also important to explore whether compounds can inhibit the action of these biomarkers or antagonize the effects of the proteins that are highly expressed in this patient subgroup.

## Conclusion

The upregulated circulating proteins in PE+ (AMBP, VTN, CLU, F2, PZP, APCS, and HBB) are involved in blood pressure regulation, extracellular matrix dynamics, and immune system function, highlighting the pathogenesis of this hypertensive disorder in the context of inflammation and immune defense. Therefore, these proteins may hold clinical value in diagnosing the severity of PE subtypes, contributing to better prevention strategies and more targeted, optimized treatments. These findings enhance our understanding of the divergent pathophysiological mechanisms between the two subgroups of PE.
